# Characterizing microbial communities associated with northern root-knot nematode (*Meloidogyne hapla*) occurrence and soil health

**DOI:** 10.3389/fmicb.2023.1267008

**Published:** 2023-11-10

**Authors:** Isaac Lartey, Gian M. N. Benucci, Terence L. Marsh, Gregory M. Bonito, Haddish Melakeberhan

**Affiliations:** ^1^Agricultural Nematology Laboratory, Department of Horticulture, Michigan State University, East Lansing, MI, United States; ^2^Department of Plant, Soil, and Microbial Sciences, Michigan State University, East Lansing, MI, United States; ^3^Department of Microbiology and Molecular Genetics, Michigan State University, East Lansing, MI, United States

**Keywords:** nematode-microbe interaction, parasitic variability, indicator species, core microbiome, soil, health

## Abstract

The northern root-knot nematode (*Meloidogyne hapla*) causes extensive damage to agricultural crops globally. In addition, *M. hapla* populations with no known genetic or morphological differences exhibit parasitic variability (PV) or reproductive potential based on soil type. However, why *M. hapla* populations from mineral soil with degraded soil health conditions have a higher PV than populations from muck soil is unknown. To improve our understanding of soil bio-physicochemical conditions in the environment where *M. hapla* populations exhibited PV, this study characterized the soil microbial community and core- and indicator-species structure associated with *M. hapla* occurrence and soil health conditions in 15 Michigan mineral and muck vegetable production fields. Bacterial and fungal communities in soils from where nematodes were isolated were characterized with high throughput sequencing of 16S and internal transcribed spacer (ITS) rDNA. Our results showed that *M. hapla*-infested, as well as disturbed and degraded muck fields, had lower bacterial diversity (observed richness and Shannon) compared to corresponding mineral soil fields or non-infested mineral fields. Bacterial and fungal community abundance varied by soil group, soil health conditions, and/or *M. hapla* occurrence. A core microbial community was found to consist of 39 bacterial and 44 fungal sub-operational taxonomic units (OTUs) across all fields. In addition, 25 bacteria were resolved as indicator OTUs associated with *M. hapla* presence or absence, and 1,065 bacteria as indicator OTUs associated with soil health conditions. Out of the 1,065 bacterial OTUs, 73.9% indicated stable soil health, 8.4% disturbed, and 0.4% degraded condition; no indicators were common to the three categories. Collectively, these results provide a foundation for an in-depth understanding of the environment where *M. hapla* exists and conditions associated with parasitic variability.

## Introduction

*Meloidogyne hapla* is a soil-dwelling plant-parasitic nematode (PPN) with a broad host range and wide distribution in diverse soils and highly variable agricultural production systems (Melakeberhan et al., 2007; Lartey et al., 2021). Moreover, *M. hapla* is one of the PPNs with parasitic variability (PV), where populations isolated from different soil types have no known genetic or morphological variation but differ in their reproductive potential to cause damage in a given plant host (Liu and Williamson, 2006; Opperman et al., 2008). The basis of PV depends on how *M. hapla* populations differ in eliciting a plant host reaction such as galling and reproductive potential (Melakeberhan et al., 2007; Lartey et al., 2021). Reproductive potential, by definition, is the total number of nematodes recovered after a predetermined period following inoculation. The soils where *M. hapla* occurs range from sandy and low in organic matter to muck soils with high organic matter, and it has been established that populations from mineral soil have higher PV than those from muck soil (Melakeberhan et al., 2010). However, the mechanisms by which *M. hapla* PV associates with soil type remain unknown.

In order to understand how *M. hapla* PV relates to soil types in agricultural production landscapes, it is important to consider soil health and the soil environment in relation to the biology of *M. hapla*. Soil health, defined as a given soil's ability to function and deliver desired ecosystem services, has biological, physicochemical, nutritional, structural, and hydrological integrity components that need to be kept in balance (Lal, 2011). Intensive cultivation and agricultural inputs in the landscapes where *M. hapla* exists have resulted in varying degrees of degradation of soil health (Lartey et al., 2021). Degradation refers to the imbalance of the soil health components diminishing a soil's ability to generate desired ecosystem services. A degraded soil environment could have positive and/or negative effects on soil organisms, and it is reasonable to assume that an organism that exists therein has to adapt to those soil health conditions (McSorley, 2003; Melakeberhan et al., 2004). Within this context, it is worth considering the biology of *M. hapla*, which has an egg, second, third, and fourth-stage juveniles, and adult stage (East et al., 2019). Eggs are laid in a gelatinous matrix completely inside a root or exposed to the soil. The second-stage juvenile, the infective stage, hatches from the egg, migrates through the soil, pierces the root with its style, establishes a feeding site, and draws nutrients from the host. The third and fourth stages are completely inside the root. The second stage juvenile has the most exposure to the soil environment. Hence, quantifying soil health is necessary in order to determine if any relationship exists between *M. hapla* presence and/or PV and soil conditions.

**Figure 1:**
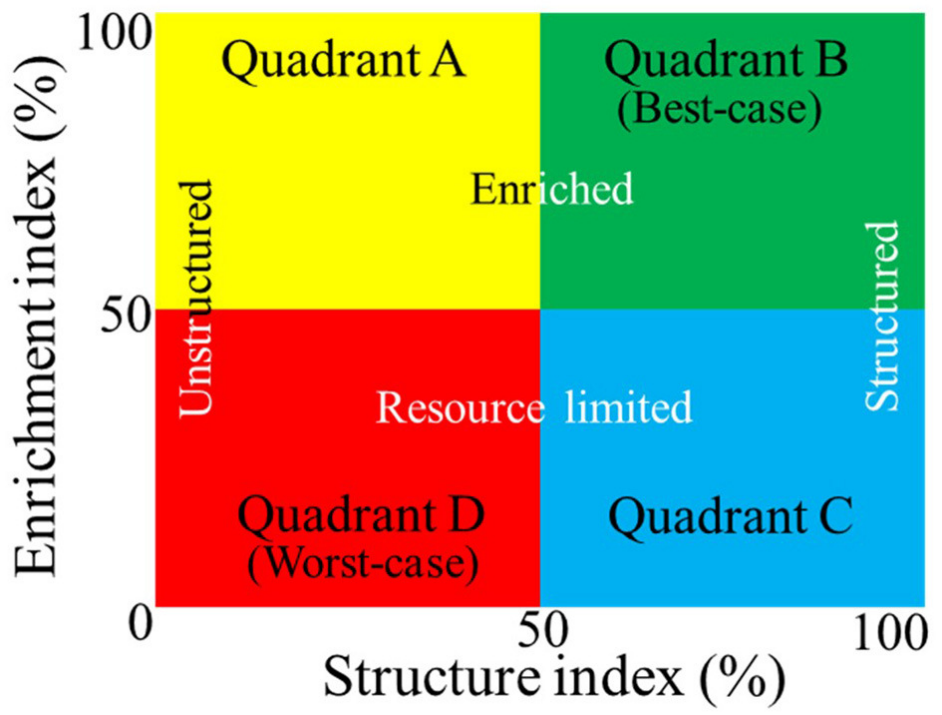
Simplified version of the Ferris et al. (2001) soil food web model using nematode bioindicators to estimate food and reproduction (Enrichment Index, vertical axis) and resistance to disturbance (Structure Index, horizontal axis). The resulting soil health categories range from the best- (Quadrant B) to worst-case (Quadrant D) scenarios for nutrient cycling and agroecosystem suitability.

Recently, Lartey et al. (2021) used the beneficial nematode community analysis-based Ferris et al. (2001) soil food web (SFW) model to map out *M. hapla* distribution in 15 mineral and muck fields in three vegetable production regions of Michigan. The SFW model uses the relationship between changes in the total nematode community dynamics in response to resource and rate of multiplication (enrichment index, EI) and resistance to disturbance (structure index, SI). The relationship between EI (y-axis) and SI (x-axis) categorizes soil conditions in terms of nutrient cycling potential and agroecosystem suitability in four quadrants from best- to worst-case scenarios (Figure 1; Ferris et al., 2001), which include the following: enriched and unstructured (Quadrant A, disturbed), enriched and structured (Quadrant B, stable, best-case), resource-limited and structured (Quadrant C), or resource-limited and minimal structure (Quadrant D, degraded, worst-case). Quadrant B describes the best and Quadrant D the worst soil health outcomes. *M. hapla* was found in soil conditions fitting the disturbed (Quadrant A) and degraded scenarios (Quadrant D) in mineral and muck fields (Lartey et al., 2021). In a follow-up study, two of the *M. hapla* populations (Fields 8 and 13) from mineral and degraded soils had significantly higher reproductive potential than the rest of the populations (Lartey et al., 2022). While confirming earlier reports that *M. hapla* populations from mineral soils have higher reproductive potential than populations from muck soils (Melakeberhan et al., 2010; Melakeberhan and Wang, 2012), the study established for the first time the soil health conditions present at locations where *M. hapla* exists and their correlation with PV.

The field observation and experimental studies prompt an overarching question: Could there be a general and/or specific connection between *M. hapla* PV and soil health—bio-physicochemical components, in particular? The roles of the soil microbiome in regulating nutrient cycling, soil health parameters (Chaparro et al., 2012; Pajares and Bohannan, 2016; Saleem et al., 2019; Sanjuan et al., 2020), and nematode-microbiome antagonistic (Chen and Dickson, 1998; Topalović et al., 2020) and mutual (Cao et al., 2015; Tian et al., 2015; Colagiero et al., 2020; Yergaliyev et al., 2021) interactions are well documented. At this stage, it is difficult to tell what direct and/or indirect relationship, if any, may exist between the presence or absence of *M hapla* and the soil microbiome and soil conditions. However, it is worth noting the occurrence of *M. hapla* in disturbed and resource-enriched (Quadrant A) and degraded and resource-depleted (Quadrant D) in mineral and muck soils and that populations with the highest reproductive potential were from mineral soils with degraded soil health conditions (Lartey et al., 2022). If there is any attributable relationship between soil health conditions and *M. hapla* PV, microbial community composition and/or structure will be a likely indicator.

Our objectives in this study were three-fold. First, we aimed to characterize the soil microbiome structure and diversity in mineral and muck soil fields with varying soil health conditions that included the presence or absence of *M. hapla*. We hypothesize that soil microbiomes would differ significantly between soil groups and soil health conditions. Our second aim was to determine the core microbiome associated with *M. hapla* occurrence across fields. Here, we define the core microbiome as the most abundant and prevalent taxa shared across the *M. hapla*-infested and non-infested fields. Knowing the status of core-microbial communities relative to the presence or absence of *M. hapla* is important to understanding the soil environment where the nematodes exist and microbes that may facilitate or interact with nematodes. We hypothesize that the core-microbial members would be present in most of the *M. hapla*-infested and non-infested fields. Our third aim was to identify indicator microbes associated with *M. hapla* occurrence and the soil health conditions as described by the SFW model (Ferris et al., 2001). The indicator microbes are distinguishable across different soil health conditions based on *M. hapla* occurrence or SFW conditions. We hypothesize that there are microbial indicators associated with *M. hapla* occurrence or SFW conditions. While earlier research of *M. hapla* populations by Liu and Williamson (2006) ruled out the correlation between pathogenicity and genetics, it is logical for our study to focus on the soil environment with a particular emphasis on soil microbial communities co-existing with *M. hapla* occurrence in different soil conditions. Collectively, the outcomes of this research will help to establish foundations for understanding the soil bio-physicochemical environment related to *M. hapla* PV.

## Materials and methods

### Sample sites with *Meloidogyne hapla* occurrence

Samples were collected from six muck and nine mineral soils in the eastern, southwestern, and northwestern vegetable production areas in the lower peninsula of Michigan, USA. The GPS coordinates of the sampled fields are shown in Supplementary Table 1. The distribution of sampled fields in the three regions and their soil health conditions as described by the SFW model (Figure 1), soil groups, and the presence or absence of *M. hapla* are graphically depicted in Supplementary Figure 1 (Lartey et al., 2021). Three each of the muck (4, 5, and 6) and mineral (1, 2, and 3) soil fields were located in the east, one muck (10) and three mineral (7, 8, and 9) fields in the southwest, and two muck (14 and 15) and three mineral (11, 12 and 13) fields in the northwest regions (Supplementary Figure 2). Fields 4, 6, and 10 (muck) and 2 (mineral) were characterized as having disturbed (Quadrant A), Fields 5, 14, and 15 (muck) and 1, 3, and 7 (mineral) as degraded (Quadrant D), and Fields 11 and 12 (mineral) as stable (Quadrant B) SFW conditions (Lartey et al., 2021). *M. hapla* was present in all muck and mineral soil Fields 2, 8, and 13 (Lartey et al., 2021). Field 13 had the highest PV population, Field 8 medium, and the rest of the fields (2, 4, 5, 6, 10, 14, and 15) registered low PV populations (Lartey et al., 2022). This study describes the relationships among soil health conditions, the presence or absence of *M. hapla*, and microbial communities in these 15 fields.

### Soil sampling and DNA extraction

A total of 75 ~1 L soil samples were collected from the top 15 cm of the 15 fields in June 2018. The samples in each field were collected from five randomly marked 25 m^2^ areas. Each sample was a composite of 10 cores collected with a custom-made 2.5 cm diameter probe (Melakeberhan et al., 2018). Each soil sample was thoroughly mixed, and a 10 ml sub-sample was collected in 15 ml falcon tubes, transported to the laboratory on ice, and stored at−80°C prior to DNA extraction.

DNA was extracted from a 2 g sub-sample of soil using the PowerSoil^®^ DNA isolation kit (Qiagen, United States). All extractions included those containing no samples (negative controls) and were stored at−80°C (Longley et al., 2020).

### Miseq library preparation and sequencing

Illumina MiSeq amplicon libraries targeting bacterial 16S rDNA with the primers 515F and 806R and fungal ITS rDNA with the primers ITS1f and ITS2 were constructed (White et al., 1990; Gardes and Bruns, 1993; Caporaso et al., 2011; Kozich et al., 2013). Libraries were prepared using Accuprime Pfx Super Mix. The polymerase chain reaction (PCR cycles used for 16S and ITS are shown in Supplementary Tables 2, 3, respectively. All PCR products were normalized with the SequalPrep Normalization Plate Kit (Thermo Fisher Scientific, United States), and the final concentration of the library was determined using a SYBR green quantitative PCR (qPCR) assay with primers specific to the Illumina adapters (Kappa). Following normalization, samples were combined into one pool and concentrated with Amicon Ultra 0.5 mL 50K filters (EMD Millipore, Germany). Libraries were then cleaned with Agencourt AMPure XP magnetic beads to remove small fragments and primer dimers (Beckman Coulter, United States). Libraries were sequenced at the MSU Genomics Core with the Illumina MiSeq v2 500 cycles kit. Sequence data generated in this study have been deposited into the NCBI SRA archive under the following accession number: PRJNA833458.

### Bioinformatic analysis

Bioinformatic analyses of 16S and ITS sequences were performed using Qiime 2 version 2019.1 (Bolyen et al., 2019). First, the sequences were analyzed for initial quality using FastQC. Due to the lower quality of the reverse reads, only forward reads were analyzed further for the ITS sequence, while both forward and reverse reads were used for 16S sequences. The “join-pairs” method of the q2-vsearch plugin was used to join the 16S sequence pairs. Afterward, both 16S and ITS library statistics were analyzed for quality distributions using the q2-quality-filter plugin. Additionally, error modeling, de-replication, and denoising of sequences were performed with the default values of the q2-deblur plugin. The primers were trimmed, and the read lengths for 16S and ITS truncated to 220 and 200 bp, respectively. The taxonomies of 16S and ITS representative sequences were assigned using Greengenes 13.9 and CONSTAX2 (Gdanetz et al., 2017; Liber et al., 2021) against the UNITE database version 04.02.2020 (Abarenkov et al., 2020), respectively.

### Statistical analyses

Data files containing operational taxonomic units (OTUs) tables, taxonomy, mapping, and OTU sequences were loaded into the R (version 4.0.2) statistical environment (R Core Team, 2020) and used to create a phyloseq object for further analysis in the *phyloseq* package (McMurdie and Holmes, 2013). Sequences belonging to non-target organisms, including Archaea, chloroplast, and mitochondria, were removed from the 16S data prior to performing the analysis (Zhang et al., 2019). The OTUs that were determined to be contaminants in the negative controls were removed with the decontam package (Davis et al., 2018). Alpha diversity (within sample diversity) was estimated for each sample following recommendations in McMurdie and Holmes (2014). It was estimated using observed richness (Simpson, 1949) and Shannon diversity (Hill, 1973) within the BiodiversityR and vegan packages (Dixon, 2003; Kindt and Coe, 2005). OTU richness and Shannon diversity were visualized for each field with boxplots in *ggplot2* (Wickham, 2016). Differences in alpha diversity means across fields were tested for statistical significance using Kruskal Wallis tests in the stats package (R Core Team, 2020). Afterward, a pairwise Wilcox test with an FDR (false discovery rate) *p*-value correction was performed on fields according to *M. hapla* occurrence in soil groups and soil health conditions. Following alpha diversity analyses, OTUs with less than five reads in a single sample were placed to zero to account for tag switching and PCR errors (Lindahl et al., 2013; Oliver et al., 2015). Stacked-bar plots for bacterial communities were created in *ggplot2* to show phyla and taxonomic classes with >2% relative abundance, while classes with <2% abundance were grouped as other (Wickham, 2016). Fungal stacked-bar plots were created to show all phyla and families with >1.5% relative abundance, while families with < 1.5% abundance were grouped as other. Next, data were normalized by cumulative sum scaling in the *metagenomeSeq* package (Paulson et al., 2013). Following normalization, beta diversity was analyzed in the *phyloseq* and *vegan* packages by creating principal coordinates analysis (PCoA) plots with the “ordinate” and “plot_ordination” functions. Here, *M. hapla* occurrence relative to soil group and region community patterns was reported. An ellipse covering 70% of data points was drawn to show clusters of *M. hapla* occurrence in soil groups. Community patterns identified in PCoA plots were tested for statistical significance using PERMANOVA as implemented by the “adonis” function in *vegan*. The homogeneity of variance between modeled groups was analyzed with the “betadisper” function in *vegan*.

The most prevalent (core) microbes were identified following the “increase method” described by Shade and Stopnisek (2019). Briefly, microbial OTUs were ranked by occupancy across fields, the proportion of total community explained by core subset taxa estimated using the Bray-Curtis method for beta-diversity, and core-taxa identified at 2% as the threshold for a marginal return in the explanatory value. The taxonomic genera of the identified core taxa were assigned using NCBI nucleotide BLAST^®^ (https://blast.ncbi.nlm.nih.gov/Blast.cgi?PAGE_TYPE=BlastSearch) and then visualized using relative abundance stacked-plots.

Taxa closely associated with (a) *M. hapla* occurrence across the fields and (b) soil health conditions, as described in Figure 1, were determined with the *indicspecies* package (De Cáceres and Legendre, 2009). Following the identification of indicator OTUs, *p*-values were FDR adjusted, and only taxa with adjusted *p* < 0.05 were considered to be indicators. The top 25 most abundant identified indicator taxa associated with *M. hapla* occurrence were used to create heatmaps displaying the relative abundance distributions across fields in the *ComplexHeatmap* package in R (Gu et al., 2016). The taxonomic genera of the top 25 indicators were assigned using NCBI nucleotide BLAST^®^ (https://blast.ncbi.nlm.nih.gov/Blast.cgi?PAGE_TYPE=BlastSearch). Indicators associated with soil health conditions were visualized with a Venn diagram. All R code and files for producing figures and tables, including metadata and OTU tables, are available at: https://github.com/larteyis/Scientific-Papers-R-Code/tree/main/Lartey_et_al_2021_Field_M.hapla_Associated_Microbiome.

## Results

To study bacteria and fungi communities associated with the northern root-knot nematode (*Meloidogyne hapla*) in Michigan, a total of 75 soil samples were collected for high-throughput amplicon sequencing. These included 6 muck and 3 mineral soils from fields infested with *M. hapla* and 6 non-infested mineral soil fields with either degraded, disturbed, or maturing soil health conditions in three regions. Each of these 75 samples was analyzed for 16S, as well as for ITS rDNA fungal diversity. A total of 3,443,432 and 1,283,828 raw sequence reads were obtained for 16S and ITS libraries, respectively. The quality filtering procedure obtained 12,906 OTUs for 16S and 2,067 OTUs for ITS (Supplementary Figure 3).

### Bacterial community composition

Only 10 phyla had >2% relative abundance. Phyla with <2% were categorized as other (Supplementary Figure 4A). The detected phyla were present at varying proportions and occurring in all fields or varying by presence or absence of *M. hapla*, soil health conditions, and/or region. Acidobacteria (9.3 to 18.3%), Actinobacteria (8.7 to 19.8%), Bacterioidetes (3.4 to 15.8%), Chloroflexi (2.9 to 9.8%), Planctomycetes, (2.2 to 6.0%), and Proteobacteria (32.3 to 52.3%) were present in soils from all fields; other Phyla accounted for 2.9 to 5.5%. Verrucomicrobia and Gemmatimonadetes were absent in disturbed SFW and *M. hapla*-infested muck Field 4 (east region) and non-infested mineral Field 7 (southwest region), respectively. Nitrospirae was present only in *M. hapla*-infested and disturbed Field 4 (east) and degraded Fields 14 and 15 (northwest) muck soils. Firmicutes were present in *M. hapla*-infested and disturbed SFW muck Field 10 (southwest), degraded mineral Fields 8 (southwest) and 13 (northwest), non-infested mineral Fields 1, 3 (east) and 7 (southwest) with degraded and Fields 11 and 12 (northwest) with stable SFW conditions (Supplementary Figure 4A).

**Figure 2:**
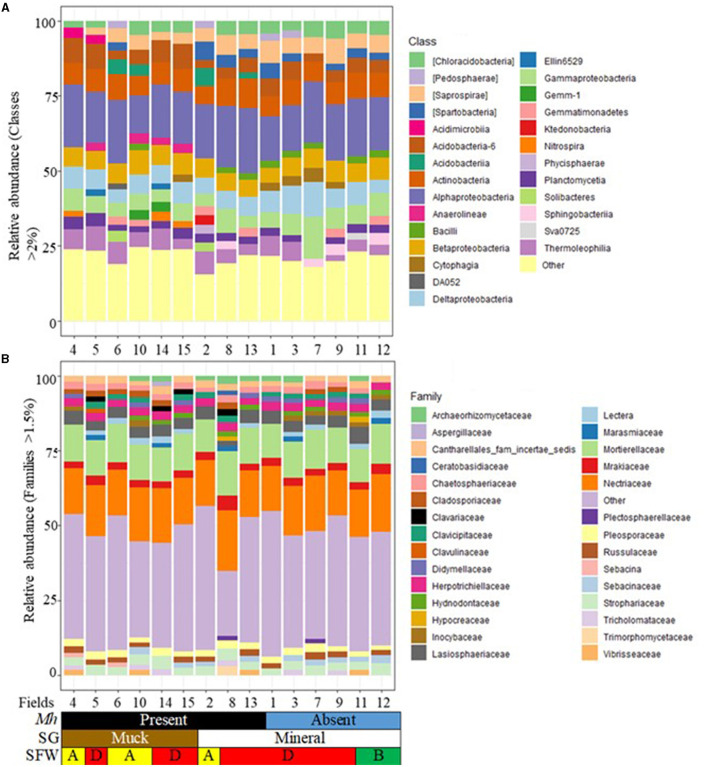
Stacked bar plots of 15 agricultural fields showing the relative abundance by *M. hapla* occurrence [*Mh*: Present (black) and Absent (blue)], soil group [SG: Mineral (white) and Muck (brown)] and soil food web conditions [SFW: D-Degraded (red), A-Disturbed (yellow), B-Stable (green)] for **(A)** bacterial classes (>2%), and **(B)** fungal families (>1.5%). The colors of bacteria and fungi correspond with colors in the stacked bar plots, and each bar represents a field. Other unassigned bacterial and fungal groups were classified as other. Relative abundance of bacterial (classes) and fungal (families) groups were variable across the 15 sampled fields.

Of the 27 classes of bacteria with more than 2% relative abundance, Alphaproteobacteria (12.7 to 21.7%), Acidobacteria-6 (2.5 to 8.6%), Actinobacteria (6.2 to 9.8%), Betaproteobacteria (5.0 to 8.0%), Deltaproteobacteria (4.3 to 11.4%), and Gammaproteobacteria (3.9 to 14.3%) and those with <2% relative abundance (15.4 to 24.7%) occurred in all fields (Figure 2A). The less prevalent classes had variable absence or presence mostly related to soil group and *M. hapla* occurrence. These included the absence of Saprospirae and Spartobacteria in one or more of the muck fields with varying soil health conditions, Chloracidobacteria in disturbed Field 6 (muck) and Field 2 (mineral), and Thermoleophilia in degraded Field 7 (mineral). The presence of Acidimicrobiia, Anaerolineae, DA052, Ellin6529, Gemm-1, and Nitrospira was limited to one or more of the muck fields, while Ktedonobacteria and Phycisphaerae were limited to mineral soil Field 2. Acidobacteriia and Solibacteres were present in one or more disturbed and/or degraded *M. hapla*-infested muck and mineral soils. Cytophagia, Gemmatimonadetes, and Pedosphaerae were present in infested and/or non-infested mineral and muck soils, whereas Sphingobacteria and Sva0725 were present in infested and/or non-infested mineral soils.

### Fungal community composition

Only 5 fungal phyla had >1% relative abundance. The phyla with <1% relative abundance were represented as other (Supplementary Figure 4B). Agaricomycetes (1.3 to 3.0%), Ascomycota (56.3 to 59.6%), Basidiomycota (26.8 to 29.4%), and Mortierellomycota (8.6 to 11.6%) and other phyla (1.2 to 3.0%) were present in all the fields. Glomeromycota (1.1 to 1.2%) were present only in *M. hapla*-infested muck fields with degraded (Fields 5) and disturbed soils (Field 6) in the eastern region and degraded mineral (Fields 13) in the northwest region and non-infested (Field 1) in the eastern region.

Twenty-nine families with at least 1.5% relative abundance were detected (Figure 2B). Cantharellales_fam_incertae_sedis (1.5 to 2.8%), Herpotrichiellaceae (2.3 to 3.1%), Lasiosphaeriaceae (1.7 to 5.6%), Mortierellaceae (10.9 to 14.7%), Mrakiaceae (2.3 to 4.9%), Nectriaceae (15.0 to 20.3%), Pleosporaceae (1.6 to 2.9%), and Strophariaceae (2.5 to 4.4%) were found in all the fields. The less prevalent families had a variable presence with no particular trend to soil group and/or *M. hapla* occurrence. For example, Chaetosphaeriaceae was absent in a non-infested and mineral (Field 12) with stable soil health conditions, while Lectera and Russulaceae were absent in infested mineral and muck soils and/non-infested mineral soils with varying soil health conditions. Aspergillaceae, Clavulinaceae, and Sebacinaceae were present in one or more muck fields with degraded and/or disturbed soil health, while Ceratobasidiaceae and Trimorphomycetaceae were present in degraded and *M. hapla*-infested mineral soil (Field 8). Hypocreaceae and Plectospaerellaceae were present in degraded and *M. hapla*-infested (Filed 8) and non-infested degraded (Field 7) and stable (Field 11) mineral soils. Archaeorhizomycetaceae, Cladosporiaceae, Clavariaceae, Clavicipitaceae, Didymellaceae, Hydnodontaceae, Inocybaceae, Marasmiaceae, Sebacinaceae, Tricholomataceae, and Vibrisseaceae had broad distribution across fields and *M. hapla* infestations (Figure 2B).

### Bacterial alpha diversity

**Figure 3:**
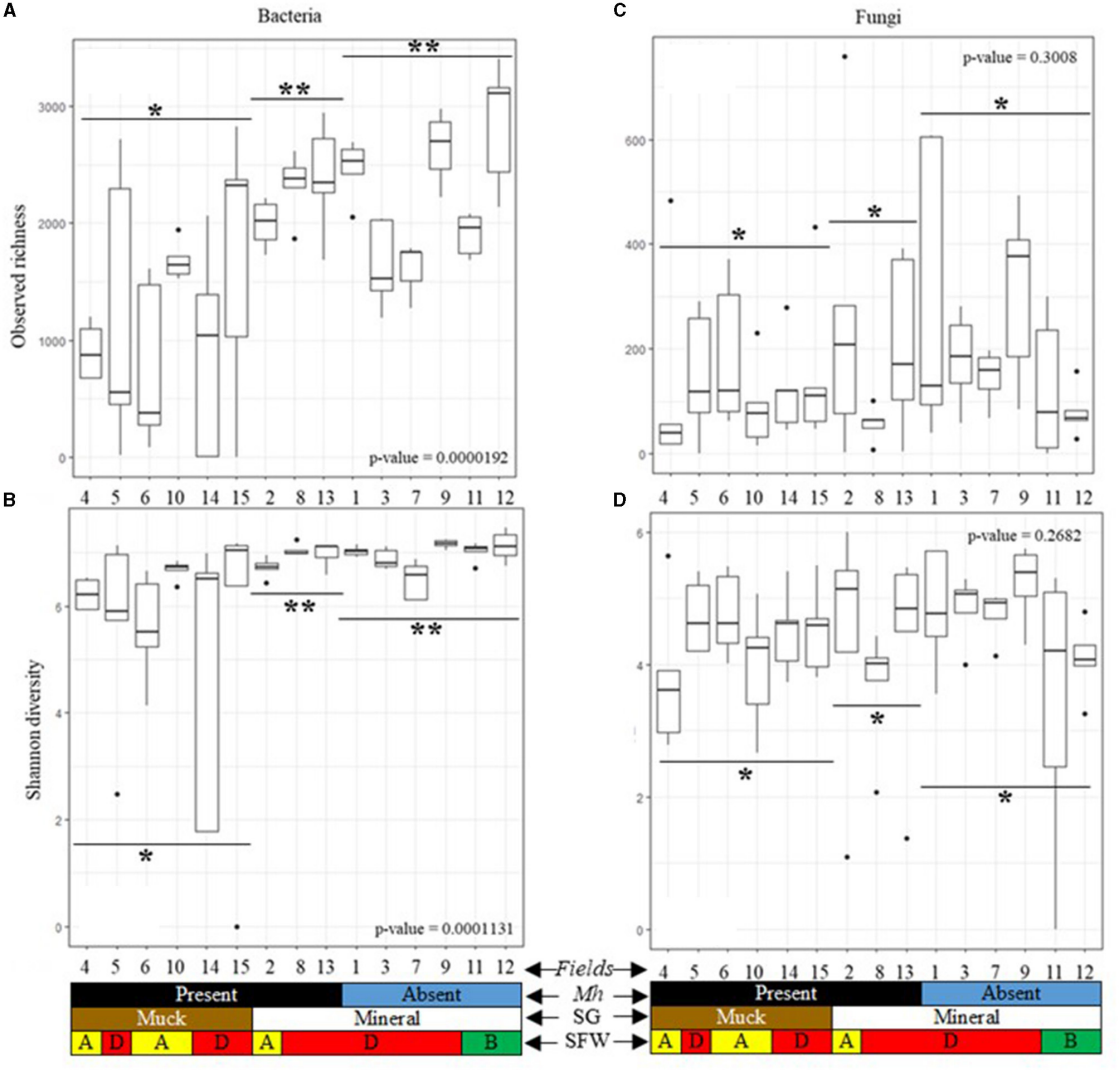
Alpha diversity boxplots of 15 agricultural fields by *M. hapla* occurrence [*Mh*: present (black) and absent (blue)], soil group [SG: mineral (white) and muck (brown)], and soil food web conditions [SFW: D-degraded (red), A-disturbed (yellow), B-stable (green)] of **(A)** bacterial observed richness, **(B)** bacterial Shannon diversity, **(C)** fungal observed richness, and **(D)** fungal Shannon diversity. Outliers on boxplots are displayed as dots. Kruskal Wallis tests were performed to determine significant differences across fields and the *p*-values shown. A pairwise Wilcox test with an FDR *p*-value correction compared alpha diversity by soil groups based on *M. hapla* occurrence (muck with *M. hapla* present, mineral with *M. hapla* present, and mineral with *M. hapla* absent). Each boxplot represents a field. Lines show groups, and the asterisk (*) symbol shows the differences/similarities of groups. Different asterisks were used to note significant differences (*p* < 0.05).

Alpha diversity of each field was measured using the observed richness and Shannon diversity. Bacterial richness and diversity differed significantly (*p* < 0.05) across all muck and mineral fields (Figures 3A, B). Observed richness in muck fields [1 to 2,825 (average 1,193)] was generally lower than in mineral fields [1,187 to 3,403 (average 2,204)]. Observed richness in *M. hapla*-infested and disturbed SFW muck Fields 4, 6, and 10, and degraded Fields 5, 14, and 15 was lower than in disturbed (Field 2) and degraded (Fields 8 and 13) mineral soil fields (Figure 3A). Shannon diversity in the muck [0.0 to 7.2 (average 5.7)] and mineral [6.1 to 7.5 (average 6.9)] fields, as well as in *M. hapla* infestation and SFW conditions, was similar to the observed richness (Figure 3B).

### Fungal alpha diversity

The observed richness of mineral [1 to 758 (average 185)] and muck soil [1 to 485 (average 138)], and the Shannon fungal diversity of mineral [0.0 to 6.0 (average 4.3)] and muck [0.0 to 5.6 (average 4.2)] were similar across soil groups (Figures 3C, D). In fields, the observed richness of fungi was similar regardless of soil groups, SFW conditions, or *M. hapla* infestation (Figure 3C). Similarly, the Shannon diversity of fungi was similar across the fields in the two soil groups, SFW conditions, or *M. hapla* infestation (Figure 3D).

### Beta diversity of bacterial communities

**Figure 4:**
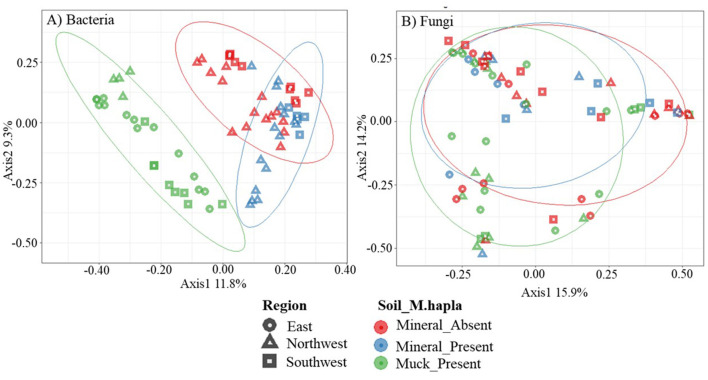
Principal coordinates analysis plots, based on Bray-Curtis dissimilarity, of **(A)** bacterial and **(B)** fungal communities. Colors represent *Meloidogyne hapla* occurrence in soil groups (mineral_absent: red, mineral_present: blue, muck_present: green), while the shapes represent the regions (east: circle, northwest: triangle, southwest: square) of the lower peninsula of Michigan. Categories are separated with a 70% ellipse.

A principal coordinate analysis (PCoA) of all field samples was used to identify patterns in bacterial communities (Figure 4A). Across soil groups, distinct separation between muck and mineral soil bacterial communities was evident along the x- (11.8%) and y-axes (9.3%). In mineral soil, *M. hapla-*infested samples shared bacterial communities with non-infested samples. The ellipse of *M. hapla*-infested mineral soil samples slightly overlapped with that of infested muck soil samples. Bacterial communities showed little graphical separations by region.

Results of PERMANOVA showed that bacterial diversity was significantly (*p* < 0.05; perm. = 9999) affected by soil groups (SG), regions (RG), SFW conditions, *M. hapla* (MH) occurrence, and their interactions (Table 1). Between 38.0 and 6.9% variation (*R*^2^) could be explained by all the variables. The homogeneity of variance showed significant group dispersion between regions (*p* < 0.05).

**Table 1:** Permutational multivariate analysis of variance *adonis* and multivariate homogeneity of groups dispersion analysis (betadisper) results for bacteria and fungi communities associated with *M. hapla* occurrence (MH), soil group (SG), region (RG), soil health (SFW) conditions, and interactions in 15 agricultural fields.

	**Bacteria**					**Fungi**				
	**Permanova**			**Dispersion**		**Permanova**			**Dispersion**	
**Variable**	***F-value***	***R** ^2^*	***p-value***	***F-value***	***p-value***	***F-value***	***R** ^2^*	***p-value***	***F-value***	***p-value***
MH	5.353	0.069	**0.0001**	43.036	**0.001**	1.495	0.02	0.0966	0.702	0.427
SG	8.368	0.104	**0.0001**	12.644	**0.001**	1.788	0.024	**0.0383**	1.045	0.349
RG	3.685	0.093	**0.0001**	0.764	0.467	1.141	0.031	0.2544	0.478	0.608
SFW	3.867	0.098	**0.0001**	14.11	**0.001**	1.198	0.032	0.1985	0.4172	0.676
SG:MH	5.928	0.142	**0.0001**	12.258	**0.001**	1.241	0.033	0.1623	0.631	0.551
RG:MH	3.647	0.173	**0.0001**	17.803	**0.001**	1.524	0.1	**0.0065**	0.303	0.918
SFW:MH	3.881	0.142	**0.0001**	14.547	**0.002**	1.172	0.047	0.1991	0.333	0.797
SG:SFW:MH	4.608	0.252	**0.0001**	8.154	**0.001**	1.241	0.083	0.0884	0.56	0.749
SG:RG:SFW:MH	3.914	0.380	**0.0001**	2.591	**0.015**	1.269	0.15	**0.0330**	0.215	0.989

Significant p-values are indicated in bold.

### Beta diversity of fungal communities

The PCoA of fungal communities did not reveal any patterns based on *M. hapla* occurrence and soil groups (Figure 4B). Approximately 30% (15.9% on the x-axis and 14.3% on the y-axis) of the total variation was accounted for. No observable separations along the x- and y-axes were noted in soil groups or regions by *M. hapla* occurrence.

The PERMANOVA showed that fungal diversity was significant (*p* < 0.05; perm = 9,999) by SG, RG^*^MH, and SG^*^RG^*^SFW^*^MH interactions, accounting for 2.4, 10, and 15% of the variation (*R*^2^), respectively (Table 1). The homogeneity of variance test did not reveal any significant differences (*p* < 0.05) within the group sample dispersion of any variable.

### Core bacterial communities

**Figure 5:**
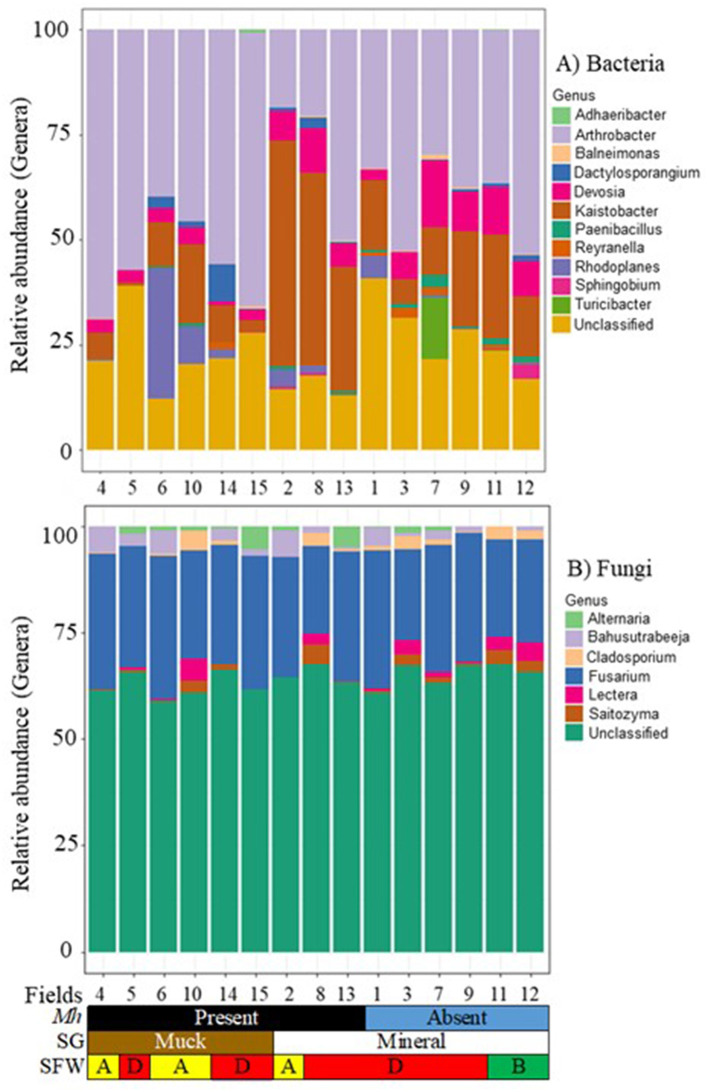
Stacked bar plots of 15 agricultural fields by *M. hapla* occurrence [*Mh*: present (black) and absent (blue)], soil group [SG: mineral (white) and muck (brown)], and soil food web conditions [SFW: D-degraded (red), A-disturbed (yellow), B-stable (green)] for core **(A)** bacterial, and **(B)** fungal communities. The colors of bacteria and fungi correspond with colors in the stacked bar plots, and each bar represents a field. Other unassigned bacterial and fungal genera were assigned as unclassified. The relative abundance of bacterial and fungal genera was variable across the 15 sampled fields.

Across all muck and mineral fields, 39 core bacterial OTUs were detected. These were classified into 11 genera (Figure 5A). *Arthrobacter* (17.8 to 67.8%), *Devosia* (1.1 to 10.0%), *Kaistobacter* (0.9 to 53.3%), and the unclassified taxa were the most common genera across fields regardless of soil groups, SFW conditions, and *M. hapla* occurrence. Other less prevalent core genera were present in both soil groups with either disturbed (Fields 2, 4, 6, and 10), degraded (Fields 1, 3, 7, 8, 9, and 13), or stable (Fields 11 and 12) and *M. hapla*-infested muck (Fields 4, 5, 6, 10, 14, and 15) and mineral (Fields 2, 8, and 13) or non-infested (Fields 1, 3, 7, 9, 11, and 12) fields. *Adhaeribacter, Balneimonas, Dactylosporangium, Paenibacillus, Reyranella, Rhodoplanes, Sphingobium*, and *Turicibacter* were variable across fields. The core bacteria included many functional groups including those associated with suppressive soil (*Arthrobacter* and *Dactylosporangium*), nematicidal (*Devosia*), enhanced nematode parasitism (*Kaistobacter*), plant growth promoter (*Paenibacillus*), soybean cyst associated (*Reyranella*), root-knot nematode associated (*Rhodoplanes*), and polysaccharide degrader (*Sphingobium*) functional groups. On the other hand, little information on *Adhaeribacter, Balneimonas*, and *Turicibacter* interaction with nematodes exists in the literature.

### Core fungal communities

Forty-four OTUs were detected as core fungal communities across muck and mineral fields and classified into 6 genera, and the unassigned OTUs were grouped as unclassified (Figure 5B). *Fusarium* (20.0 to 33.4%) was common in all fields regardless of soil groups, SFW conditions, and *M. hapla* presence. As part of the core fungi, *Alternaria, Bahusutrabeeja, Lectera*, and *Saitozyma* varied in soil groups, SFW conditions, and *M. hapla* infestation. The core fungi were plant pathogenic (*Fusarium, Alternaria*, and *Lectera*) and polysaccharide degrader (*Saitozyma*) functional groups. However, little is known about the function of *Bahusutrabeeja*.

### Indicators of *M. hapla* occurrence and soil health conditions

**Figure 6:**
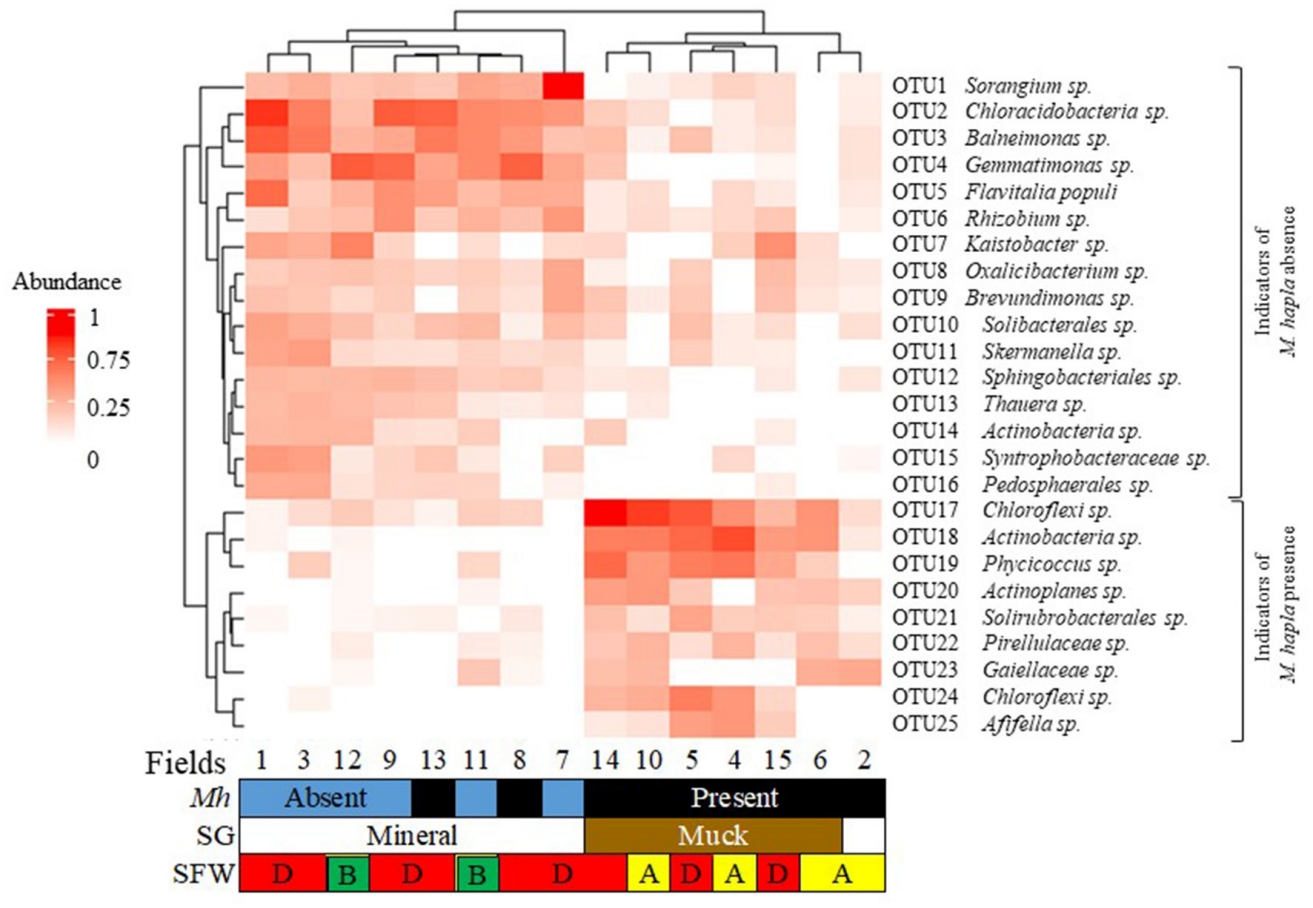
Bacterial indicator heatmap of the top 25 most abundant sub-operational taxonomic units (OTUs) associated with *M. hapla* occurrence [*Mh*: present (black) and absent (blue)] across soil groups [SG: mineral (white) and muck (brown)] and soil food web conditions [SFW: D-degraded (red), A-disturbed (yellow), B-stable (green)]. A deeper red color corresponds with a higher bacteria abundance. On the y-axis (hierarchical clustering) is the lower cluster showing indicators associated with *M. hapla* presence (OTU17 to OTU25), and the upper cluster (Bray-Curtis dissimilarity) showing indicators of *M. hapla* absence (OTU1 to OTU16). The lower cluster with taxa such as *Chloroflexi* and *Actinobacteria* was a strong indicator of *M. hapla* presence in muck soils and the mineral Field 2. The top cluster with taxa such as *Sorangium* and *Chloracidobacteria* was a strong indicator of mineral fields with and without *M. hapla*. Fields 13 and 8 were associated with the high and medium PV and separated from the low PV populations.

A heatmap of the top 25 most relatively abundant indicator bacterial OTUs associated with the occurrence of *M. hapla* in all muck and mineral fields is shown in Figure 6. The indicator OTUs associated with *M. hapla* occurrence were clustered by hierarchical clustering on the y-axis, whereas fields were clustered by Bray-Curtis dissimilarity on the x-axis. All mineral soil Fields (8 and 13 and 1, 3, 7, 9, 11, and 12), but Field 2, clustered separately from all of the muck soil fields. Accordingly, the relatively abundant OTUs in mineral fields were OTU1 to OTU16. The relatively abundant OTUs in the muck Fields 4, 5, 6, 10, 14, and 15 and mineral Field 2, accordingly, were OTU17 to OTU25. On the y-axis, the relatively more abundant OTUs associated with *M. hapla* presence formed a separate cluster from the OTUs associated with *M. hapla* absence.

The functional groups of the abundant indicator bacteria (OTU1 to OTU16) in the mineral soils were the following: *Sorangium* (polysaccharide degrader); *Chloracidobacteria* (nematicidal); *Balneimonas* and *Gemmatinomonas* (suppressive soil)*; Flavitalia populi* (plant pathogenic); *Rhizobium* (nitrogen fixer); *Kaistobacter* (enhanced nematode parasitism); *Brevundimonas* and *Solibacterales* (plant growth promoter); *Sphingobacteriales* (root-knot nematode associated); and *Oxalicibacterium, Skermanella, Thauera, Actinobacteria, Syntrophobacteraceae*, and *Pedosphaerales* (other). The functional groups of the relatively less abundant indicator bacteria (OTU17 to OTU25) in the muck Fields and mineral Field 2 were *Chloroflexi* (OTU17 & 24); *Actinobacteria; Solirubrobacterales* (nematicidal); *Actinoplanes* (suppressive soil); *Afifella* (root-knot nematode associated); *Phycicoccus, Pirellulaceae* and *Gaiellaceae* (other).

**Figure 7:**
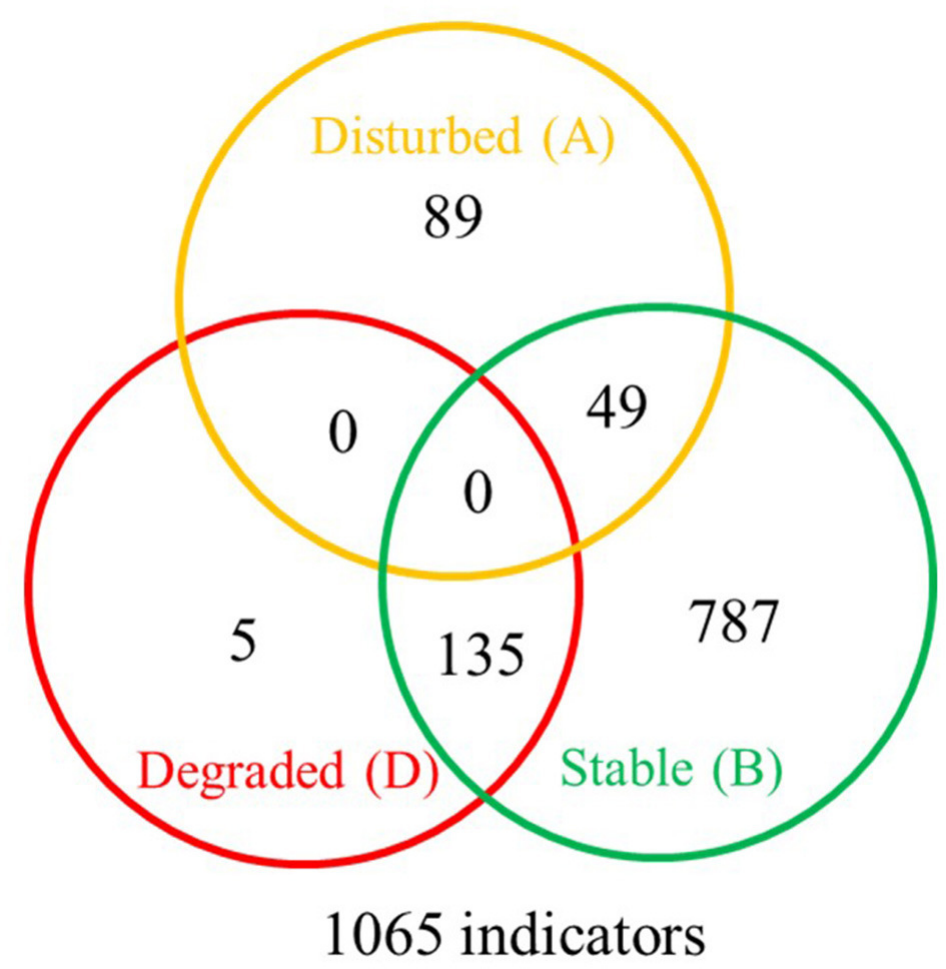
Venn diagram showing the distribution of 1,065 bacterial indicator sub-operational taxonomic units (OTUs) associated with disturbed (A-yellow), degraded (D-red), and stable (B-green) soil health conditions. The degraded soil health conditions had 5 unique indicators, the disturbed had 89, and the maturing ones had 787 indicators. The stable conditions shared 135 indicators with degraded and 49 indicators with disturbed conditions. No indicators were shared by degraded and disturbed conditions or by all three soil health conditions. A complete list of soil health condition indicators is available at https://github.com/larteyis/PAPER-Bacterial-composition-diversity-and-functional-groups-associated-with-Meloidogyne-hapla-popul/blob/cb0d1614156b4fc2092e495274ba42ce88d31716/SFW_ISA_LIST.xlsx%20-%20Sheet1.pdf.

The SFW conditions were disturbed (Fields 2, 4, 6, and 10), degraded or worst case (Fields 1, 3, 7, 8, 9, and 13), and stable or best case (Fields 11 and 12). A total of 1,065 indicator OTUs were associated with the disturbed (yellow), degraded (red), and stable (green) categories of soil health conditions (Figure 7). Of these, 5 OTUs were specific to degraded, 89 OTUs to disturbed, and 787 OTUs to stable soil health conditions. Soils with degraded SFW conditions had 135 OTUs, and those with disturbed conditions shared 49 OTUs with soils that had stable conditions. There were no OTUs shared between disturbed and degraded and among disturbed, degraded, and stable soil health conditions.

## Discussion

A growing body of research indicates that *M. hapla* populations from different soil conditions exhibit parasitic variability (PV) (Melakeberhan et al., 2007; Melakeberhan and Wang, 2013; Lartey et al., 2022), but little is known about how PV relates to the bio-physicochemical environment in general and any role of soil microbiome, in particular. This study was designed to develop baseline information toward understanding associations among soil microbiome, soil health, and *M. hapla* occurrence.

### Microbial diversity and community composition

The first aim of this study was to characterize the soil microbiome structure and diversity in mineral and muck fields that varied in soil health conditions and *M. hapla* occurrence. Our hypothesis that soil microbiome would differ significantly between soil groups (SG) and soil health conditions (SFW) was partially supported by the results. We found that bacterial community composition varied by SG, region (RG), SFW, and *M. hapla* occurrence (MH), and that of fungi varied by SG x RG x SFW x MH interactions. Lower bacterial diversity (observed richness and Shannon) observed in muck fields with more disturbed and degraded soil health conditions than in *M. hapla*-infested or non-infested mineral soil with similar degradations also supports our hypothesis. In contrast, the lack of difference in diversity and richness of fungi by RG, SFW, or MH, but SG, does not support the hypothesis. Together, this suggests that a more limited set of factors influences fungal community structure in contrast to bacterial composition, which had several drivers influencing the community structure in the soil. Perhaps this explains the differences observed in bacterial diversity and the lack of difference in fungal diversity.

The variable effects of soil type and/or region on bacterial and fungal community composition and/or diversity are consistent with published literature (Fierer and Jackson, 2006; Lupatini et al., 2013; Zhou and Fong, 2021). In addition, our results revealed that the abundance of commonly occurring bacterial and fungal communities present in all fields or some fields varied by SG, RG, SFW, and/or MH occurrence. For example, Proteobacteria and Alphaproteobacteria and fungal phyla Ascomycota and Basidiomycota were present in high proportions and in all fields (Supplementary Figure 4; Figure 2). In contrast, bacterial Verrucomicrobia and 19 out of 27 classes of bacteria, and the fungal phylum Glomeromycota and 22 out of 29 families had low relative abundance (Supplementary Figure 4; Figure 2).

As seen in Supplementary Figure 2, *M. hapla* was present in 3 mineral (Fields 2, 8, and 13) and 6 muck (Fields 4, 5, 6, 10, 14, and 15) soil fields with degraded (Fields 5, 8, and 13) and disturbed (Fields 2, 4, 6, and 10) soil health conditions, and the *M. hapla* populations from these fields were characterized as high (Field 13), medium (Field 8) and low (Fields 2, 4, 5, 6, 10, 14, and15) PV (Lartey et al., 2022). In this regard, it is more informative to look at the absence or presence and functions of the bacterial and fungal communities relative to the PV of *M. hapla* populations and soil health conditions. Our results reveal several trends within and across PV categories and soil health conditions.

First, the absence or presence of bacterial communities and their functions in soil from where the low PV *M. hapla* populations came show a mixed environment. Some Verrucomicrobia species are endosymbionts of some species of *Xiphinema americanum* group, and their function could be related to an improvement of nematode nutrient uptake from plants (Brown et al., 2015; Zhou et al., 2019). Gemmatimonadetes (common in root-knot nematode suppressive soils) (Zhou et al., 2019), Saprospirae and Chloracidobacteria (common in root-knot nematode infested and non-infested soils) (Zhou et al., 2019), and Spartobacteria (associated with the corky-root disease complex in tomatoes) (Lamelas et al., 2020) were not detected in one or more of the low PV fields with either disturbed and/or degraded soil health conditions (Supplementary Figure 4; Figure 2). On the other hand, the presence of Nitrospirae (suppresses root-knot nematode infection) and Acidimicrobiia (common in root-knot nematode infested and non-infested rhizosphere) were limited to one or more of the muck fields, and Ktedonobacteria (with nematicidal properties against the root-knot nematode affecting soybeans) (Toju and Tanaka, 2019) and Phycisphaerae (associated with root-knot nematode infection) to mineral soil Field 2 (Figure 2A). It is currently difficult to determine the impact of mostly root-knot–suppressing bacterial communities on the soil from where the low PV populations came.

Second, many of the bacterial and fungal communities present in *M. hapla*-infected mineral and muck soil fields appear to be associated with some form of parasitism on nematodes (Figure 2). These include the presence of bacterial Acidobacteriia (common in root-knot nematode-infested and non-infested rhizosphere) (Zhou et al., 2019) and Solibacteres (negative abundance relationship with *Meloidogyne* spp.) (Castillo et al., 2017; Zhou et al., 2019), and fungi Aspergillaceae (involved in the pine wilt disease vectored by the nematode *Bursaphelenchus xilophilus*) (Vicente et al., 2021), Clavulinaceae (multi-functional), and Sebacinaceae (includes an indicator of the absence of the pine wilt disease) (Liu et al., 2021) in both soils. Meanwhile, the fungi families Ceratobasidiaceae (involved in the pine wilt disease vectored by the nematode *Bursaphelenchus xilophilus*) (Vicente et al., 2021) and Trimorphomycetaceae (have nematicidal properties against the root-knot nematode affecting soybeans) (Toju and Tanaka, 2019) were limited to mineral soils (Figure 2).

Third, bacteria and fungi with broad distribution across soil health categories and the presence of *M. hapla*. These include the presence of Firmicutes (parasitic bacteria) (Hussain et al., 2018) in *M. hapla*-infested disturbed muck (Field 10) and degraded (Fields 8 and 13) and non-infested mineral (Fields 1, 3, 7, 11, and 12) soils; fungi Hypocreaceae (involved in the pine wilt disease vectored by the nematode *Bursaphelenchus xilophilus*) (Vicente et al., 2021); and Plectospaerellaceae (parasitic bacteria) (Carlucci et al., 2012) in *M. hapla*-infested (Field 8) and non-infested degraded (Field 7) and stable (Field 11) mineral soils. However, not much is known about DA052 (Field 6), Ellin6529 (Fields 5 and 14), Gemm-1 (Fields 10 and 14), and Acidobacteriia (Fields 2, 6, 10, and 13).

Finally, there are the bacteria and fungi communities that vary across SG, RG, SFW, and MH. These include bacterial such as Cytophagia (known to occur in both infested and non-infested root-knot nematode soils) (Zhou et al., 2019) and Sphingobacteriia (negatively impacts *Caenorhabditis elegans* population numbers) (Dirksen et al., 2016) (Figure 2A) and fungi such as Sebacinaceae (antagonistic to cyst nematode infection and development in *Arabidopsis* roots) (Daneshkhah et al., 2013) and Tricholomataceae (negatively affects the activity of the pinewood nematode, *B. xilophilus*) (Ishizaki et al., 2015) (Figure 2B).

These results suggest that bacterial and fungi groups were widely distributed regardless of soil conditions, *M. hapla* occurrence, or geography. Whether or not any of the fungal and bacterial functional groups have any relationship to *M. hapla* PV is yet to be determined.

### Core microbial communities

Our second aim was to identify the core soil microbiome associated with the occurrence of *M. hapla* across fields using the abundance-occupancy distributions as suggested by Shade and Stopnisek (2019) We identified 39 bacterial and 44 fungal microbes as core microbes present in at least most of the *M. hapla*-infested and non-infested fields, supporting our hypothesis. *Fusarium*, a ubiquitous and diverse fungal genus, and the bacterial genera *Arthrobacter, Devosia*, and *Kaistobacter*, as well as unclassified taxa, were present in all sampled fields. *Arthrobacter* includes decomposers (Cacciari and Lippi, 2009), *Kaistobacter* appears to suppress bacterial wilt (Liu, 2016), and *Devosia* has a soil toxin degrading (Talwar et al., 2020), as well as antagonistic traits against the plant-parasitic nematodes *Pratylenchus neglectus, M. chitwoodi*, and *Globodera pallida* (Eberlein et al., 2016; Castillo et al., 2017). If the core microbiome is related to PV, it is reasonable to assume that the variable taxa, or their relative abundances, may be involved.

The presence of the rest of the 39 bacteria and 44 fungi core OTUs in the fields across the three regions varied by soil group and *M. hapla* occurrence. The *M. hapla* populations in Fields 2, 8, and 13 were from mineral soil, and those in Fields 4, 5, 6, 10, 14, and 15 were from muck soil. Field 13, had an *M. hapla* population with significantly higher reproductive potential than Field 8, and both from the rest of the fields (Lartey et al., 2022). Whether or not the core microbial populations contribute to *M. hapla* PV is yet to be determined, but their presence or absence is worth noting. Field 13 where the highest reproductive potential *M. hapla* population came from lacked the fungus *Alternaria* and the bacteria *Reyranella* and *Rhodoplanes*. On the other hand, Field 8 where the medium PV population came from lacked bacteria *Paenibacillus* and *Reyranella* and the fungus *Lectera. Paenibacillus* is a beneficial bacteria that enhances plant growth through nitrogen fixation and phosphate and potassium solubilization (Patowary and Deka, 2020). *Reyranella* is found to be associated with *Heterodera glycines* (Hu et al., 2017), and *Rhodoplanes* has a positive relationship with *M. incognita* (Castillo et al., 2017). *Alternaria* is a pathogen of the citrus black rot disease (Umer et al., 2021), and *Lectera* is a legume pathogen (Cannon et al., 2012). The detection of the other core bacteria and fungi in mineral and muck soils infected with one or more of the respective populations suggests that the *M. hapla* populations were exposed to a common microbiome.

With regards to soil health conditions, the bacteria *Balneimonas, Dactylosporangium, Rhodoplanes*, and *Sphigobium* and the fungi *Bahusutrabeeja, Lectera*, and *Saitozyma* were common to the disturbed and degraded muck and mineral soils. The bacteria *Paenibacillus* was detected in disturbed and degraded mineral and disturbed muck soils, while the fungus *Cladosporium* was found in disturbed mineral and disturbed and degraded muck soil. Although there is little information about *Bahusutrabeeja* and *Balneimonas* in the literature, other core microbes associated with soil health conditions had different roles. *Dactylosporangium* is found in *Heterodera glycines* suppressive soils (Topalović et al., 2020), whereas *Saitozyma* is a yeast (Li et al., 2020), and *Sphingobium* produces enzymes that allow sugars to be degraded (Balows et al., 1992; Wu et al., 2017). *Cladosporium* is involved in the increase of systemic defense in pine to reduce the incidence of *Bursaphelenchus xylophilus* infectivity (Chu et al., 2019). While the role of the core microbes reported in this study relative to *M. hapla* PV is unknown, documenting their presence or absence is helpful in understanding the SFW conditions in which *M. hapla* exists.

### Indicator bacterial communities relative to m. hapla occurrence and soil health conditions

The third aim of this study was to identify indicator microbes associated with *M. hapla* occurrence and SFW conditions. We identified 25 bacterial OTUs serving as indicators for *M. hapla* presence (dominated by OTU17 to OTU25) or absence (dominated by OTU1 to OTU16) and 1,065 OTUs serving as indicators of soil health conditions. None of the 1,065 soil health indicator OTUs were common to the three categories—disturbed, degraded, or steady. The clustering of the bacterial OTUs by *M. hapla*, soil group, and soil health conditions and the 1,065 OTUs by soil health categories partially support the hypothesis that there are indicator microbes associated with *M. hapla* occurrence or soil health conditions (Figures 6, 7). While the enrichment of bacterial indicator species in the fields was variable, Field 2 clustered with the muck fields, all of which were *M. hapla* infested and separately from the rest of the mineral soil fields. This suggests that there may be soil-specific factors driving the indicator species. Regardless of SFW conditions, the infested muck soils were clustered and shared *Chloroflexi* sp. (OTU17), *Actinobacteria* sp., *Phycicoccus* sp., *Solirubrobacterales* sp., and *Pirellulaceae* sp. *Chloroflexi* and *Solirubrobacterales* are part of a consortium of anti-nematode bacteria in the rhizosphere of soybean plants attacked by root-knot nematodes (Toju and Tanaka, 2019), and *Actinobacteria* introduced to strawberry root by *Pratylenchus penetrans* cause a decline in strawberry yield. However, little is known about the role of *Phycicoccus* and *Pirellulaceae*.

Although *M. hapla* populations in Field 2 had similar PV as those from muck soil (Lartey et al., 2022), the reason behind its clustering with muck soils is unknown. The fields where *M. hapla* populations with highest PV (Fields 13 and 8), and Field 2 came from had *Sorangium* sp., *Chloracidobacteria* sp., *Balneimonas* sp., *Gemmatinomonas* sp., *Flavitalia populi, Rhizobium* sp., *Oxalicibacterium* sp., *Solibacterales* sp., *Sphingobacteriales* sp., *Chloroflexi* sp. (OTU17), and *Solirubrobacterales* sp. in common. Yet, Fields 8 and 13 were distinct from Field 2 and all other populations from muck soil. It is unknown if the enrichment of *Actinoplanes* sp. and *Actinobacteria* sp. (OTU18) in Field 2, and their absence in Field 8, along with the presence of *Actinobacteria* sp. (OTU14) and *Pedosphaerales* sp. in Field 13, contributed to the similarity and differences in the clustering of these populations. Additionally, degraded soil health conditions in the latter two fields and disturbed soil health conditions in the former field may have played a role in this as well. *Sorangium* has enzymes that break down plant cell walls (Li et al., 2013), and *Chloracidobacteria* is part of the microbiome in the rhizosphere known to suppress root-knot nematode infection (Zhou et al., 2019). *Balneimonas* is associated with amendment-treated soils that suppress verticillium wilt (Inderbitzin et al., 2018), *Gemmatinomonas* and *Actinoplanes* are present in plant parasitic nematode suppressive soils (Topalović et al., 2020), and *Flavitalia* is associated with apple replant disease (Kanfra et al., 2022). *Rhizobium* fixes nitrogen in plants to enhance growth (Maróti and Kondorosi, 2014), *Solibacterales* enhance plant growth by mobilizing phosphorus in the soil (Bergkemper et al., 2016), and *Sphingobacteriales* is associated with *M. incognita* infection in tomato roots (Tian et al., 2015). The functions of *Oxalicibacterium* and *Pedosphaerales* are not known. Whether the shared indicators have a relationship with PV or not is also not fully understood.

*Meloidogyne hapla* was isolated in mineral and muck soils with disturbed, degraded, and stable soil health conditions (Lartey et al., 2021), and populations from Fields 13 and 8 from degraded mineral soil had significantly higher PV than the rest of the populations in both soil health categories (Lartey et al., 2022). Of the 1,065 indicator bacterial species found across the soil health categories, 73.9% were in the stable, 8.4% in the disturbed, and 0.4% in the degraded soil conditions, supporting generally known facts about soil degradation relative to microbial communities (Doran and Zeiss, 2000). The soils with stable conditions shared 8.4% of indicator OTUs with disturbed and 12.7% with degraded soil health conditions, suggesting that there was some commonality between the soil health conditions. As summarized in Figure 6, OTU indicator species of *M. hapla* were primarily associated with the bacterial phyla Chloroflexota and Actinomycetota, while soils lacking *M. hapla* were indicated by OTUs belonging to more diverse phyla including Myxoccota, Acidobacteriota, Gemmatimonadota, Pseudomonadata, Bacterioidata, Themodesulfobacteriota, and Verrucomicrobiota. However, there are limitations when interpreting the interactions and how they may relate to *M. hapla* and microbial communities. For example, *M. hapla* was present in muck and only in one-third of the mineral soil fields, and the effects of SG, SFW, and RG on microbial communities were greater than the presence or absence of *M. hapla* (Table 1). The lack of common indicator OTUs between the disturbed and the degraded soil health categories and/or *M. hapla* presence or absence suggests that the conditions in these soil health groups are conducive for different microbes. Whether all or some of the soil microbes are directly, indirectly, or of no consequence relative to PV demands further investigation. However, characterizing the microbial communities in soils where *M. hapla* occurs relative to PV is an important first step in knowing how the soil environments influence survival and interactions therein.

Characterizing the soil bio-physicochemical environment where *M. hapla* populations with varying degrees of PV is the first step toward understanding the mechanisms of its PV. This study provides the first insights into the occurrence of *M. hapla* in mineral and muck soil groups with disturbed and degraded soil health conditions. It also offers fresh insights into their association with microbial community structure and function and core- and indicator-microbiomes across three regions in the lower peninsula of Michigan, USA. Although the OTUs/taxa identified may be biased to the green genes database, taken together, these findings provide a basis for further exploration of how the microbial communities may or may not be related to *M. hapla* PV.

## Data availability statement

The datasets presented in this study can be found in online repositories. The names of the repository/repositories and accession number(s) can be found in the article/Supplementary material.

## Author contributions

IL: Data curation, Formal analysis, Methodology, Validation, Visualization, Writing—original draft. GMNB: Writing—review & editing, Formal Analysis. TM: Investigation, Writing—review & editing. GB: Investigation, Supervision, Writing—review & editing. HM: Writing—review & editing, Conceptualization, Funding acquisition, Project administration, Supervision.
