# Supplementary material for: Characterizing microbial communities associated with northern root-knot nematode (Meloidogyne hapla) occurrence and soil health
Source: Front Microbiol. 2023 Nov 10;14:1267008. doi: 10.3389/fmicb.2023.1267008 (PMC10667709; doi:10.3389/fmicb.2023.1267008)
Supplement: Supplementary file 1 [file Data_Sheet_1.docx]

**Characterizing microbial communities associated with the northern root-knot nematode (*Meloidogyne hapla*) occurrence and soil health**

**Isaac Lartey^1,4^, Gian MN. Benucci^2^, Terence L. Marsh^3^, Gregory M. Bonito^2^ and Haddish Melakeberhan^1*^**

*^1^Agricultural Nematology Laboratory, Department of Horticulture, Michigan State University, East Lansing, MI 48824, USA*

*^2^Department of Plant, Soil, and Microbial Sciences, Michigan State University, East Lansing, MI 48824, USA*

*^3^Department of Microbiology and Molecular Genetics, Michigan State University, East Lansing, MI 48824, USA*

*^4^Current Address: Westat Inc., 1600 Research Blvd. Rockville, MD 20850, USA*

* **Correspondence:**

Haddish Melakeberhan

Email: [melakebe@msu.edu](mailto:melakebe@msu.edu)

**Keywords:** Nematode-microbe Interaction, Parasitic variability, Indicator species, Core microbiome, Soil health

**Abstract**

The northern root-knot nematode (*Meloidogyne hapla*) causes extensive damage to agricultural crops globally. In addition, *M. hapla* populations with no known genetic or morphological differences exhibit parasitic variability (PV) or reproductive potential associated with soil type. However, why *M. hapla* populations from mineral soil with degraded soil health conditions had a higher PV than populations from muck soil is unknown. To improve understanding of soil biophysicochemical conditions in the environment where *M. hapla* populations exhibited PV, this study characterized the soil microbial community, core- and indicator- species structure associated with *M. hapla* occurrence and soil health conditions in 15 Michigan mineral and muck vegetable production fields. Bacterial and fungal communities in soils from where nematodes had been isolated were characterized with high throughput sequencing of 16S and Internal Transcribed Spacer (ITS) rDNA. Results showed that, *M. hapla*-infested, as well as disturbed and degraded muck fields, had lower bacterial diversity (observed richness and Shannon) compared to corresponding mineral soil fields or non-infested mineral fields. Bacterial and fungal community abundance varied by soil group, soil health conditions and/or *M. hapla* occurrence. A core microbial community was found to consist of 39 bacterial and 44 fungal sub-operational taxonomic units (OTUs) across all fields. In addition, 25 bacteria were resolved as indicator OTUs associated with *M. hapla* presence or absence, and 1,065 bacteria as indicator OTUs associated with soil health conditions. Out of the 1,065 bacterial OTUs, 73.9% were indicator of the stable, 8.4% of the disturbed, 0.4% of the degraded condition, and no indicators were common to the three categories. Collectively, these results provide a foundation for in-depth understanding of the environment where *M. hapla* exists and conditions associated with PV.

**SUPPLEMENTARY**

**Table S1 |** Field coordinates of 15 agricultural fields sampled.

| **Field** | **Latitude** | **Longitude** |
| --- | --- | --- |
| **1** | 43.068871° | -83.341871° |
| **2** | 43.092822° | -83.053522° |
| **3** | 43.071761° | -83.032147° |
| **4** | 43.065446° | -83.067139° |
| **5** | 43.050561° | -83.068175° |
| **6** | 43.094628° | -83.064396° |
| **7** | 42.664865° | -86.047077° |
| **8** | 42.108973° | -86.368160° |
| **9** | 42.083001° | -86.367671° |
| **10** | 42.660808° | -85.996822° |
| **11** | 43.840772° | -86.348123° |
| **12** | 43.826522° | -86.378970° |
| **13** | 43.764887° | -86.137222° |
| **14** | 43.351260° | -85.726828° |
| **15** | 43.197608° | -85.782483° |

**Table S2 |** PCR mix and thermocycle used for PCR

| **PCR MIX** |  |  |  | **THERMOCYCLE** |  |  |
| --- | --- | --- | --- | --- | --- | --- |
|  | | |  |  | | |
| **PCR Mix** | **µL** | **Total (µL)** |  | **Temp (°C)** | **Time(min)** | **Cycles** |
| Dream Taq | 6.25 | 937.5 |  | 95 | 2:00 |  |
| ITS1F | 0.375 | 60 |  | 95 | 0:20 | 30x |
| ITS2R | 0.375 | 60 |  | 55 | 0:15 |  |
| H20 | 2 | 320 |  | 72 | 5:00 |  |
| BSA | 2 | 320 |  | 72 | 10:00 |  |
| DNA | 1 |  |  | 4 | inf |  |

**Table S3 |** PCR mix and thermocycle used for 3-step PCR

| **PCR MIX** |  |  |  | **THERMOCYCLE** |  |  |
| --- | --- | --- | --- | --- | --- | --- |
| **Step 1** | | |  | **Step 1** | | |
| **PCR Mix** | **µL** | **Total (µL)** |  | **Temp (°C)** | **Time(min)** | **Cycles** |
| Dream Taq | 6.25 | 937.5 |  | 95 | 5:00 |  |
| ITS1F | 0.375 | 60 |  | 95 | 0:30 | 10x |
| ITS2R | 0.375 | 60 |  | 50 | 0:30 |  |
| H20 | 2 | 320 |  | 72 | 1:00 |  |
| BSA | 2 | 320 |  | 72 | 7:00 |  |
| DNA | 1 |  |  | 12 | inf |  |
|  |  |  |  |  |  |  |
| **Step 2** |  |  |  | **Step 2** |  |  |
| **PCR Mix** | **µL** | **Total (µL)** |  | **Temp (°C)** | **Time(min)** | **Cycles** |
| Dream Taq | 6.25 | 1000 |  | 95 | 5:00 |  |
| ITS1F (with frameshift) | 0.375 | 60 |  | 95 | 0:30 | 10x |
| ITS2R (with frameshift) | 0.375 | 60 |  | 50 | 0:35 |  |
| H20 | 1 | 160 |  | 72 | 1:05 |  |
| BSA | 2 | 320 |  | 72 | 7:00 |  |
| DNA | 2 from step1 |  |  | 12 | inf |  |
|  |  |  |  |  |  |  |
| **Step 3** |  |  |  | **Step 3** |  |  |
| **PCR Mix** | **µL** | **Total (µL)** |  | **Temp (°C)** | **Time(min)** | **Cycles** |
| Dream Taq | 8 | 1280 |  | 95 | 5:00 |  |
| PCR F | 0.5 | 80 |  | 95 | 0:40 | 10x |
| H20 | 0.5 | 80 |  | 63 | 0:50 |  |
| Barcode | 1 |  |  | 72 | 7:00 |  |
| DNA | 4 from step2 |  |  | 72 | 7:00 |  |
|  |  |  |  | 12 | inf |  |

**
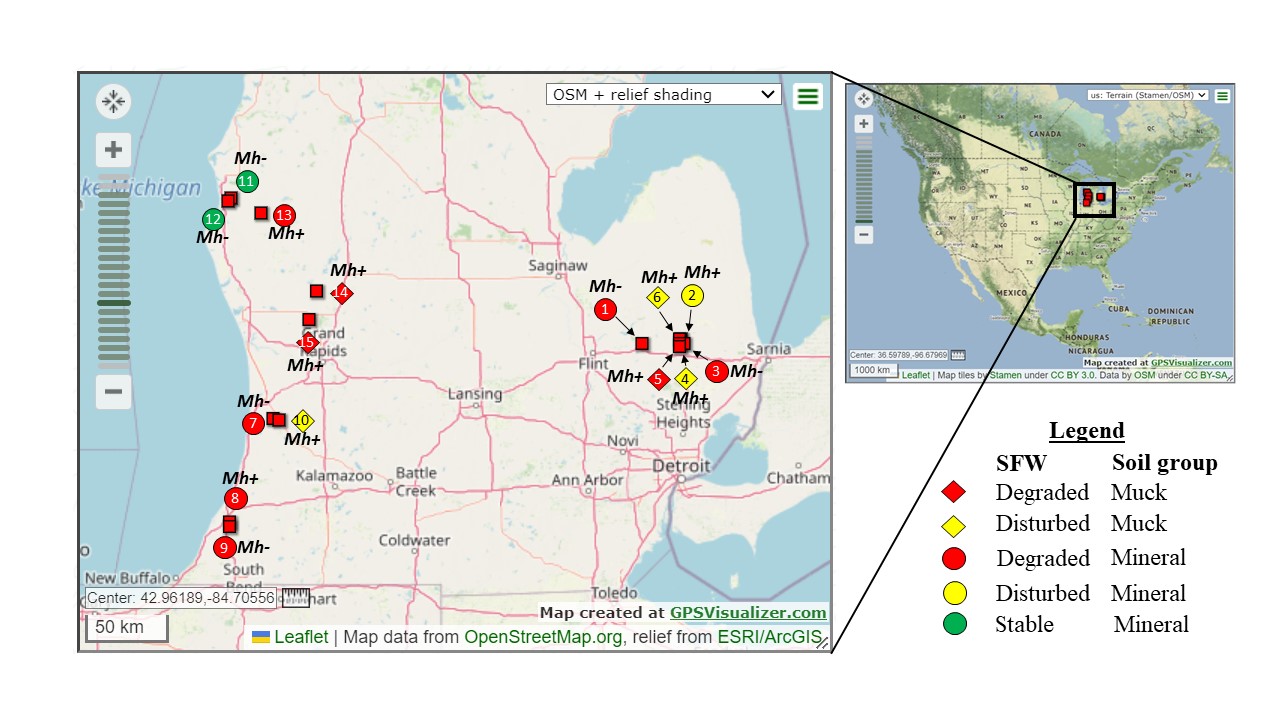
**

**Fig. S1**| Location of 15 sampled agricultural fields showing *M. hapla* occurrence (Present [Mh+] and Absence [Mh-]), soil group (Mineral [circle] and Muck [diamond]) and soil food web conditions (SFW: Degraded [red], Disturbed [yellow], Stable [green])

**Fig. S2** | Description of the 15 agricultural fields showing *M. hapla* occurrence (Mh: Present [black] and Absent [blue]), soil group (SG: Mineral [white] and Muck [brown]), soil food web conditions (SFW: D-Degraded [red], A-Disturbed [yellow], B-Stable [green]), and regions (E-Eastern [grey], S-Southwestern [purple] and N-Northwestern [orange]).


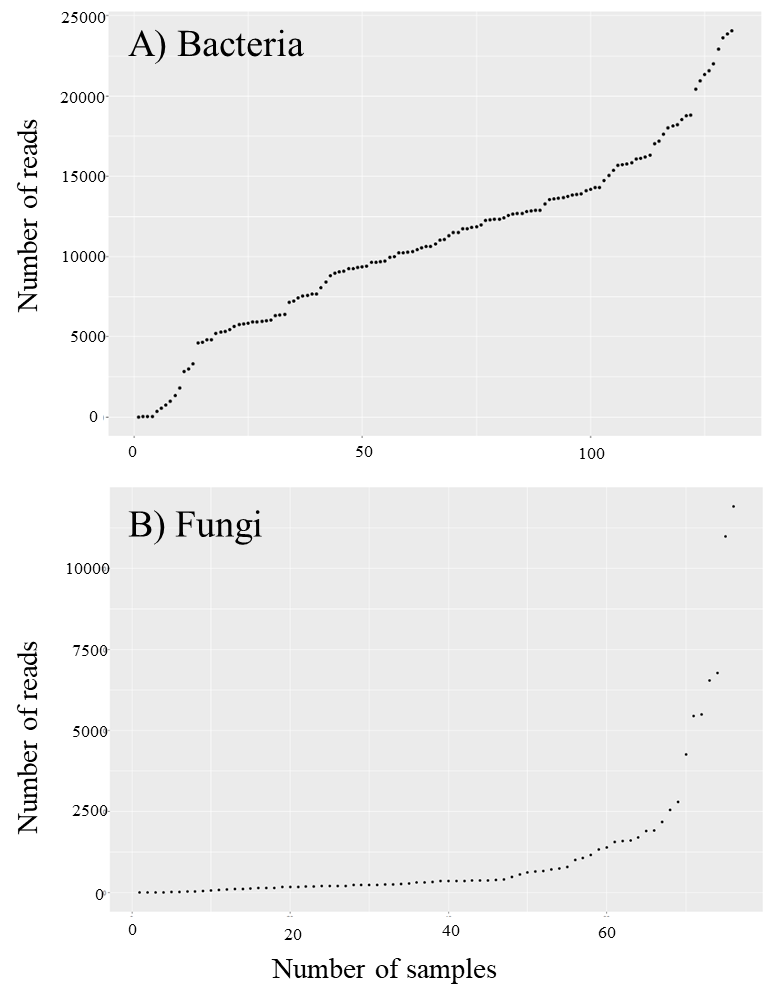


**FIG.S3 |** Number of (A) bacteria and (B) fungi reads that passed QC per sample.

**
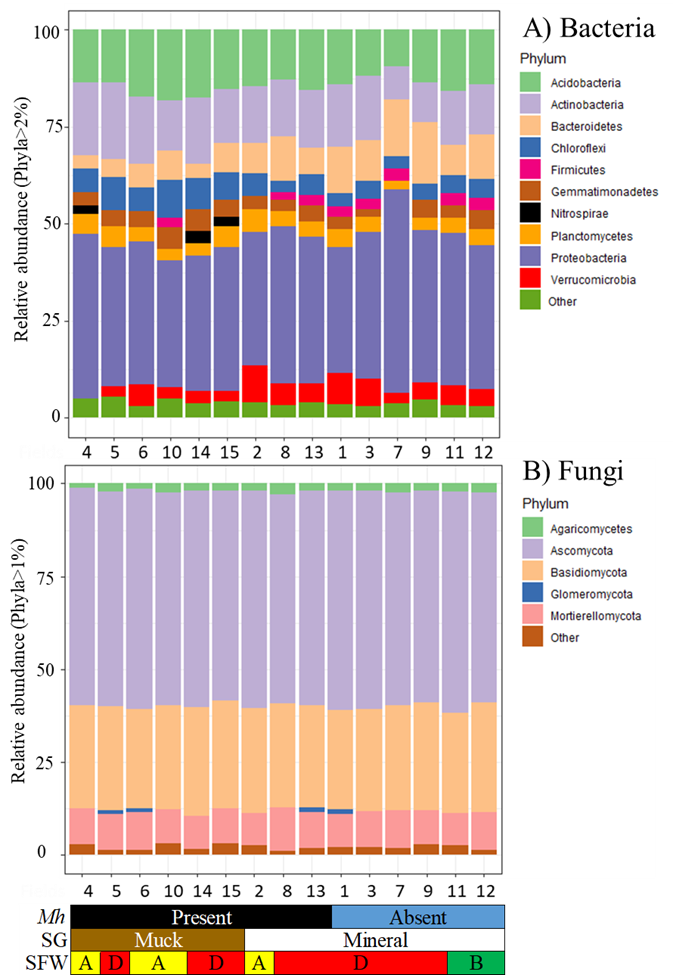
**

**FIG.S4 |** Stacked bar plots of 15 agricultural fields by *M. hapla* occurrence (Mh: Present [black] and Absent [blue]), soil group (SG: Mineral [white] and Muck [brown]) and soil food web conditions (SFW: D-Degraded [red], A-Disturbed [yellow], B-Maturing [green]) of (A) bacteria phyla, and (B) fungi phyla. Colors of bacteria and fungi correspond with colors in the stacked bar plots and each bar represents a field. Other unassigned bacterial and fungal phyla were classified as other. Relative abundance of bacterial and fungal phyla variable across the 15 sampled fields.
